# Energy-Aware Dynamic DU Selection and NF Relocation in O-RAN Using Actor–Critic Learning

**DOI:** 10.3390/s22135029

**Published:** 2022-07-03

**Authors:** Shahram Mollahasani, Turgay Pamuklu, Rodney Wilson, Melike Erol-Kantarci

**Affiliations:** 1 School of Electrical Engineering and Computer Science, University of Ottawa, Ottawa, ON K1N 6N5, Canada; sh.mollahasani@gmail.com (S.M.); turgay.pamuklu@uottawa.ca (T.P.); 2 Ciena, Ottawa, ON K2K 0L1, Canada; rwilson@ciena.com

**Keywords:** actor–critic learning, energy-efficiency, O-RAN, RAN optimization

## Abstract

Open radio access network (O-RAN) is one of the promising candidates for fulfilling flexible and cost-effective goals by considering openness and intelligence in its architecture. In the O-RAN architecture, a central unit (O-CU) and a distributed unit (O-DU) are virtualized and executed on processing pools of general-purpose processors that can be placed at different locations. Therefore, it is challenging to choose a proper location for executing network functions (NFs) over these entities by considering propagation delay and computational capacity. In this paper, we propose a Soft Actor–Critic Energy-Aware Dynamic DU Selection algorithm (SA2C-EADDUS) by integrating two nested actor–critic agents in the O-RAN architecture. In addition, we formulate an optimization model that minimizes delay and energy consumption. Then, we solve that problem with an MILP solver and use that solution as a lower bound comparison for our SA2C-EADDUS algorithm. Moreover, we compare that algorithm with recent works, including RL- and DRL-based resource allocation algorithms and a heuristic method. We show that by collaborating A2C agents in different layers and by dynamic relocation of NFs, based on service requirements, our schemes improve the energy efficiency by 50% with respect to other schemes. Moreover, we reduce the mean delay by a significant amount with our novel SA2C-EADDUS approach.

## 1. Introduction

The next generation wireless networks include a fundamental transformation which is the next generation radio access networks (NG-RAN) [1]. The NG-RAN protocol stack can be split into eight different disaggregated options which are combined within three network units: radio unit (RU), distributed unit (DU), and centralized unit (CU). Furthermore, unlike the traditional RAN, NG-RAN functions can be virtualized on top of general-purpose hardware. In Open RAN (O-RAN), the concept of virtualized RAN (vRAN) and the disaggregation of network units reaches its full interoperability by using open interfaces among multiple vendors [2,3]. However, the placement of the virtual network functions can be challenging due to multiple constraints, such as routing path, RAN protocol splits, bandwidth limitations, maximum latency tolerance requirements, heterogeneous computational resources, and so on. The main objective of this work is to develop an RL-based network function placement scheme in a way that the energy consumption is minimized while the expected quality of service (QoS) for that network function is satisfied. In this work, resource block (RB) scheduling is considered as a network function (NF). The proposed scheme dynamically relocates this NF among DUs based on their location and processing power by targeting minimum energy consumption and satisfying the QoS requirements of users.

The idea of disaggregation in O-RAN is allowing RAN functions to be placed at different computing devices in a distributed manner [4]. Therefore, it is vital to identify how this disaggregationcan be executed and what metrics need to be satisfied to run these disaggregated components correctly. By considering the concept of function splits in O-RAN, the requirements for network functions such as minimum bit rate and the maximum latency for communication among O-RAN components (RU, DU, and CU) need to be satisfied. This work is developed based on the O-RAN architecture and split option 7.2 where the functional split is between RU and DU [1,5].

Since the amount of energy consumption in DUs is proportional to the time they are active, i.e., processing tasks, mobile network operators (MNO) are seeking an intelligent assignment model where RUs use minimum required resources at DUs, while QoS requirements such as packet delivery ratio and latency are satisfied [6]. Note that a part of DU energy consumption is fixed due to the cooling system, idle power, etc. Therefore, reducing the active time of DUs can decrease the overall energy consumption in the network. This can be achieved by optimizing load distribution among DUs with respect to their processing power and their location and setting redundant DUs to sleep mode. The main focus of this paper is to highlight the effect of deploying an AI-enabled network function relocation in a dynamic environment on energy consumption and network performance. To this end, we examined our model by considering resource block scheduling as a network function and applied our AI-based framework on it. We evaluate this model by generating User Datagram Protocol (UDP) and Transmission Control Protocol (TCP) packets (video and ITS) with different delay budgets and QoS requirements.

In this paper, we propose an RL-based resource block allocation in 5G and DU selection in O-RAN architecture. In the proposed model, we employed energy awareness as a key performance indicator and provide a multi-agent actor–critic (A2C) method as the primary novelty of this study. In this work, we employed two agents; one is responsible for allocating RB to UEs by considering the type and priority of packets, while the other agent is integrated for reducing the energy consumption by considering processing power, capacity, and propagation delay among DUs in the network. Furthermore, performance evaluation includes a mixed-integer linear programming (MILP) solver comparison to determine the gap between this novel approach and the lower bound of that multi-objective minimization problem. Thus, we demonstrate the feasibility of this advanced machine learning implementation in a disaggregated RAN scheme.

In our prior work [7], we developed an A2C-based algorithm for invoking NFs at the CU or DU by considering traffic requirements. This work extends [7] by considering the propagation delay between O-RAN components and the energy consumption, and in addition, we consider DUs with different processing capacities. The proposed model is examined for a different number of UEs, and it is compared with a heuristic model developed in our previous works and two other recent works. Our results show that the proposed model can reduce energy consumption by 50% with respect to models where network functions are executed locally under the given settings. Apart from energy conservation, the proposed model can reduce the overall mean delay for an intelligent transport system (ITS) and video traffic down to 7 ms and 12 ms, respectively. In contrast, the packet delivery ratio for ITS and video traffics will be increased up to 80% and 30%, respectively.

The rest of this paper is organized as follows: In Section 2, we discuss the recent works in this area. In Section 3, we formulate the problem and propose an MILP solution. The A2C-based algorithm is comprehensively explained in Section 4. In Section 5, the proposed scheme is compared with four baseline algorithms, and in Section 6, we conclude the paper.

## 2. Related Work

Energy-efficiency algorithms for traditional RANs have been comprehensively presented in [8]. Most of the works covered in [8] are based on on-off techniques for BSs. Some of these models rely on traffic prediction to estimate low traffic intervals, while others use cell zooming techniques to expand the BSs’ coverage with respect to their neighbors [9,10,11]. These studies consider RAN equipment as a monolithic equipment.

Energy efficiency is also considered in centralized RAN (C-RAN), where just the radio unit of BSs is disaggregated at remote radio heads (RRHs), and the rest is implemented at the baseband unit (BBU). For instance, in [12], BBU placement across physical machines in a cloud site is formulated as a bin-packing problem, and the authors tackle this problem by proposing a heuristic algorithm. Additionally, in [13,14], the authors improve the work in [12] by proposing a BBUs virtualization technique with a linear computational complexity order that reduces the power consumption in the network. Furthermore, in [15], the authors show how traffic forecasting at each BS can be used in dynamically switching (on/off) RRHs. Moreover, ref. [16] shows an end-to-end energy-efficient model by activating and placing virtual network functions (VNFs) on physical machines and distributing the traffic among them.

The aforementioned C-RAN scenarios mainly reflect the fixed functional split, and they require high fronthaul bitrate and consequently incur high deployment cost. To this end, the impact of the flexible function split is examined in several recent studies [17]. For instance, in [18], savings in power and computational resources with respect to different function splits are analytically modeled. In [19], the authors aim to optimize the energy efficiency and the midhaul bandwidth in C-RAN.

Different from these works, in this paper we evaluate the energy consumption for dynamic NF migration among edge clouds, i.e., not only CU allocation as in C-RAN but also DU allocation in O-RAN is considered. Finally, refs. [20,21] show that the migration of NFs has a non-negligible impact on energy consumption, which has not been addressed in previous works. Since we aim to address the placement of resource allocation function using machine learning, it is important to give a brief overview on the existing studies on RL-based resource allocation [22]. For instance, an RL-based resource block allocation technique is employed in a vehicle-to-vehicle network in [23].

In [24], an RL-based algorithm is proposed for optimizing the energy consumption and cost in a disaggregated RAN. Moreover, in [25], the authors develop an RL-based user-beam association and resource allocation scheme using transfer reinforcement learning. In [26], a deep RL-based resource block allocation is introduced in which RBs are allocated in a way that the mean delay is reduced. Another Deep RL approach is proposed in [27] to solve a two-tier resource allocation problem in a standalone base station. In our previous work [28], we developed an RL-based NF to schedule URLLC and eMBB packets with respect to the delay budget and channel conditions of UEs in the network. We also developed an RL-based algorithm for invoking NFs at the CU or DU by considering traffic requirements [7]. However, unlike [7], in this paper we develop a comprehensive scheme that considers the propagation delay between O-RAN components and the energy consumption, and in addition we consider DUs with different processing capacities. Furthermore, in [29], we introduced an optimization-based solution for the DU selection problem under delay constraints. This paper extends [29] by modeling the problem as a multi-objective minimization problem for jointly addressing energy-efficiency and delay.

Unlike previous works, in this paper we develop an actor–critic based DU selection scheme for a RAN with disaggregated layers (such as O-RAN) to dynamically relocate network functions among available DUs (edge servers) by considering their processing power and propagation delay in a way that the overall energy consumption in the network is reduced, while packet delivery ratio and delay budget of user traffic are satisfied.

## 3. System Model

**Figure 1:**
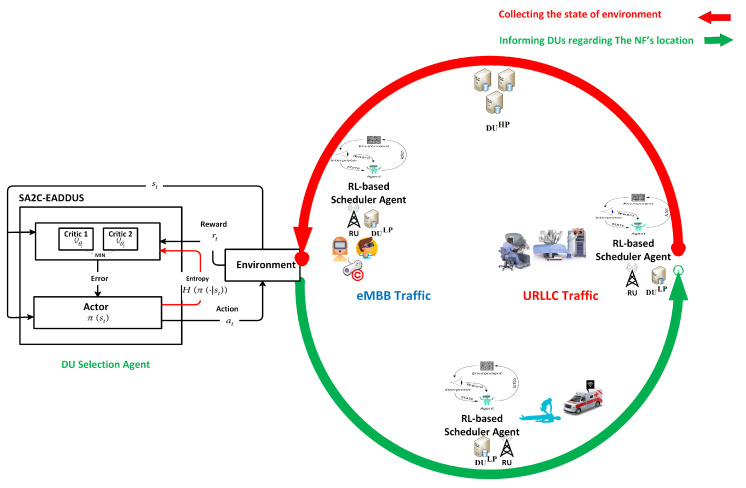
The overall architecture of the proposed model. Agents are located at DUs and interact with the DU selection algorithm by sending some feedback during each time interval (the red arrow). Then the DU selection scheme informs agents (the green arrow) regarding the location of NF during the next time interval based on the types of packets (URLLC or eMBB), available processing capacity, scheduling delay, and propagation delay among DUs to minimize the overall energy consumption in the network.

Figure 1 shows the overall architecture that is structured as a time-interval-based model. In each time interval (t∈T), *I* numbers of user equipment (UEs, i∈I) request demands which are defined as a tuple 〈i,t〉. These demands may belong to one of two (K=2) different types of traffic (k∈K), such as video and ITS. These traffic types have different demand sizes ($U_{\langle i,t\rangle k}$) and delay budgets ($\Delta_{\langle i,t\rangle k}$). On the infrastructure side, we consider *L* low processing power DUs ($DU^{LP}$, l∈L) that service these UEs. These DUs can house network protocols from the lowest level to the packet data convergence protocol [30]. Moreover, each one has a dedicated RU to perform lower layer RF functions.

Moreover, in our architecture, DUs have an option to migrate NFs to a DU with higher processing power ($DU^{HP}$). This migration option has two essential benefits in our system. First, we can switch off the digital units in the local DUs and save energy. Second, aggregation of NFs from multiple DUs in a common $DU^{HP}$ allows the resource allocation function to observe multiple RB maps in the same DU and mitigate inter-cell interference. This can lead to a lower scheduling delay and reduce the number of retransmissions in the network. However, it should be noted that $DU^{HP}$ has a limited processing capacity (ξ), and due to its location, migrating NFs to it may face higher propagation delay ($D_{l}^{P}$) with respect to other DUs, which can negatively affect packets with a lower latency delay budget. The details of the notations are given in Notations.

### 3.1. Energy Consumption and Delay Models

Equation (1) calculates the energy consumption in $DU^{LP}$*l* in time interval $t^{\prime }$. That value equals zero when NFs are migrated to $DU^{HP}$ in this time interval ($b_{lt^{\prime }}=1$) (Note that a DU may need to consume energy for other reasons in that time interval. However, these energy consumptions do not change with the decision variables; thus, we do not include them in the energy consumption model. Meanwhile, they can be easily added as constant energy consumption.). The equation provides that case by the rightmost multiplicand ($1-b_{lt^{\prime }}$). Processing NFs has two energy consumption terms; the first one is the fixed energy consumption ($E_{l}^{F}$), which does not change by the activity of the processing unit in this DU. The second one is the dynamic energy consumption which is considered when the processing unit is active for user traffic demand. Thus, it depends on the processing unit energy consumption per RB ($E_{l}^{D}$) and the DU utilization that is calculated by the number of allocated RBs *r* in the time interval $t^{\prime }$. Here $a_{\langle i,t\rangle rt^{\prime }}$ equals one if the traffic demand 〈i,t〉 is processed in RB *r* in the time interval $t^{\prime }$. Lastly, Equation (2) calculates the total energy consumption in DUs.

(1) $$E_{lt^{\prime }}=(E_{l}^{F}+E_{l}^{D}*\sum_{\langle i,t\rangle \in \langle I,T\rangle \;\;r\in R}a_{\langle i,t\rangle rt^{\prime }})*\left(1-b_{lt^{\prime }}\right)$$

(2) $$E^{TOT}=\sum_{t^{\prime }\in T}(E^{HP}+\sum_{l\in L}E_{lt^{\prime }})$$

The delay of each traffic demand (〈i,t〉) is the summation of two terms in which the first one is the scheduling delay ($\delta_{\langle i,t\rangle }^{S}$), and the second one is the propagation delay ($\delta_{\langle i,t\rangle }^{P}$). Equation (3) calculates the scheduling delay in which $y_{\langle i,t\rangle t^{\prime }}$ is a binary decision variable equaling ‘1’, if the traffic demand 〈i,t〉 scheduled/assigned to process in an RB in time interval $t^{\prime }$. However, for a large traffic demand, RBs in a single time interval may not be enough to finish that demand; thus, multiple time intervals might be assigned to demand 〈i,t〉. For that reason, we find the most recent assigned time interval ($\max_{t^{\prime }\in T}\left(y_{\langle i,t\rangle t^{\prime }}*t^{\prime }\right)$) for that demand (〈i,t〉). Then, we subtract the demand time interval (*t*) from that value to find the number of time intervals waited for that demand (〈i,t〉). Finally, we multiply that value with the length of transmission time interval (TTI) to find the scheduling delay.

(3) $$\delta_{\langle i,t\rangle }^{S}=(\max_{t^{\prime }\in T}\left(y_{\langle i,t\rangle t^{\prime }}*t^{\prime }\right)-t)*TTI$$

(4) $$\delta_{\langle i,t\rangle }^{P}=\frac{D_{M(i)}^{P}}{\sum_{t^{\prime }\in T}y_{\langle i,t\rangle t^{\prime }}}\sum_{t^{\prime }\in T}y_{\langle i,t\rangle t^{\prime }}*b_{M\left(i\right)t^{\prime }}$$

(5) $$\delta^{M}=\frac{\sum_{\;k\in K\;\;\langle i,t\rangle \in \langle I,T\rangle }\left(\delta_{\langle i,t\rangle }^{S}+\delta_{\langle i,t\rangle }^{P}\right)*u_{\langle i,t\rangle k}}{\sum_{\;k\in K\;\;\langle i,t\rangle \in \langle I,T\rangle }u_{\langle i,t\rangle k}}$$

Equation (4) defines the propagation delay for demand 〈i,t〉. As explained, a user demand may be scheduled for more than one time interval ($y_{\langle i,t\rangle t^{\prime }}>1$). Some of these time intervals may belong to the times that NFs processed in $DU^{LP}$ ($b_{M\left(i\right)t^{\prime }}=0$) (M(i) is a given value that maps the user *i* with its $DU^{LP},\left(l=M\left(i\right)\right)$.). In those time intervals, propagation delay equals zero due to negligible distance between UEs and $DU^{LP}$. On the other hand, in some intervals, NFs may be processed in $DU^{HP}$ ($b_{M\left(i\right)t^{\prime }}=1$). To calculate the number of time intervals in the latter case, we first multiply the scheduled time intervals with the NF migration decision variable ($y_{\langle i,t\rangle t^{\prime }}*b_{M\left(i\right)t^{\prime }}$). That value will equal one if and only if NFs are migrated to $DU^{HP}$ in time interval $t^{\prime }$. Second, we divide that value by the total time intervals needed to process that demand ($\sum_{t^{\prime }\in T}y_{\langle i,t\rangle t^{\prime }}$). That value gives us the percentage of time we process that demand in $DU^{HP}$. Finally, we multiply that value with the propagation delay between $DU_{l}^{LP}$ and $DU^{HP}$ ($D_{M(i)}^{P}$) to find the total propagation delay to finish that user demand.

Equation (5) shows the mean delay in the network. Here, the dividend is the total delay, and the divisor is the number of total demand in the network. In addition, $u_{\langle i,t\rangle k}$ is the traffic demand indicator, which equals one if there is a demand (〈i,t〉) in type *k*; otherwise, it equals zero.

### 3.2. System Constraints

Equation (6) guarantees that each UE gets its service demand from the system. Equation (7) prevents the allocation of the same RB ($rt^{\prime }$) to multiple user traffic demands (〈i,t〉). Equation (8) correlates the $y_{\langle i,t\rangle t^{\prime }}$ decision variable with $a_{\langle i,t\rangle rt^{\prime }}$, indicating that at least one resource block r∈R in time interval $t^{\prime }$ is allocated to the user demand 〈i,t〉. Thus, we can simplify our delay calculation by using $y_{\langle i,t\rangle t^{\prime }}$ instead of a more complex decision variable $a_{\langle i,t\rangle rt^{\prime }}$. Equation (9) generates the user traffic indicator $u_{\langle i,t\rangle k}$ from demand size $U_{\langle i,t\rangle k}$. A UE may demand only one type of traffic in a specific time interval which Equation (10) ensures. In addition, the total number of $DU_{l}^{LP}$ that can migrate their NFs to $DU^{HP}$ is limited by ξ in Equation (11). Lastly, the delay of each demand is limited by $\Delta_{\langle i,t\rangle k}$ according to their traffic type *k* in Equation (12).

(6) $$\begin{array}{cc} \sum_{t^{\prime }=t}^{T}\sum_{r\in R}S_{irt^{\prime }}*a_{\langle i,t\rangle rt^{\prime }}\geq \sum_{k\in K}\left(U_{\langle i,t\rangle k}*u_{\langle i,t\rangle k}\right), & \;\forall \langle i,t\rangle \in \langle I,T\rangle \end{array}$$

(7) $$\begin{array}{cc} \sum_{\langle i,t\rangle \in \langle I,T\rangle }a_{\langle i,t\rangle rt^{\prime }}\leq 1, & \;\forall l\in L,\forall r\in R,\forall t^{\prime }\in T \end{array}$$

(8) $$\begin{array}{cc} M*y_{\langle i,t\rangle t^{\prime }}-\sum_{r\in R}a_{\langle i,t\rangle rt^{\prime }}\geq 0, & \;\forall \langle i,t\rangle \in \langle I,T\rangle ,\forall t^{\prime }\in T \end{array}$$

(9) $$\begin{array}{cc} M*u_{\langle i,t\rangle k}-U_{\langle i,t\rangle k}\geq 0, & \;\forall \langle i,t\rangle \in \langle I,T\rangle ,\forall k\in K \end{array}$$

(10) $$\begin{array}{cc} \sum_{k\in K}u_{\langle i,t\rangle k}\leq 1, & \;\forall \langle i,t\rangle \in \langle I,T\rangle \end{array}$$

(11) $$\begin{array}{cc} \sum_{l\in L}b_{lt}\leq \xi , & \;\forall t\in T \end{array}$$

(12) $$\begin{array}{cc} \delta_{\langle i,t\rangle }^{S}+\delta_{\langle i,t\rangle }^{P}\leq \sum_{k\in K}u_{\langle i,t\rangle k}*\Delta_{\langle i,t\rangle k}, & \;\forall \langle i,t\rangle \in \langle I,T\rangle \end{array}$$

### 3.3. Problem Definition

We have a multi-objective minimization (P1) problem in which we aim for a balance between the energy consumption in DUs and the mean delay with the objective function (Equation (13)). *W* is a weighting factor between these key performance indicators (KPIs) in this equation, and Ω is a scaling factor to normalize the ranges.

(13) $$\;\left(P1\right)\;\mathrm{Minimize}:\;W*E^{TOT}+\Omega *\left(1-W\right)*\delta^{M}$$

Subject to: Equations (6)–(12)

Let us consider a special case of the problem (P1) in which the propagation delay, $D_{l}^{P}$, between any $DU^{LP}$ and $DU^{HP}$ is remarkably huge and makes the network choose only $DU^{LP}$ for NFs. Thus, P1 reduces to an RB allocation problem, which is proved as an NP-hard problem by Yu et al. [31]. Therefore, we propose an actor–critic solution for this problem which is explained in the next section.

## 4. Actor–Critic Solution

### 4.1. RL Model

In this work, we employ two nested A2C agents which are developed based on O-RAN architecture. While one agent is responsible for scheduling resource blocks during each time interval, the other is designed to dynamically choose a proper DU for executing the scheduler agent by considering energy consumption, processing power, scheduling, and propagation delay with respect to each DU’s traffic load and location. Processing resource block allocation decisions of multiple RUs on a single DU ($DU^{HP}$) can expand the observation level of decision agents, which can lead to applying more accurate actions. More precisely, when an A2C-based scheduler agent can access other RUs’ resource block map, subcarriers can be allocated to edge UEs in a way that the inter-cell interference among RUs are minimized as shown in [28]. The goal of the proposed actor–critic agent is improving the performance of the A2C-based scheduler and reducing the overall energy consumption by dynamically selecting DUs by considering their processing power and propagation delay in the network.

**Figure 2:**
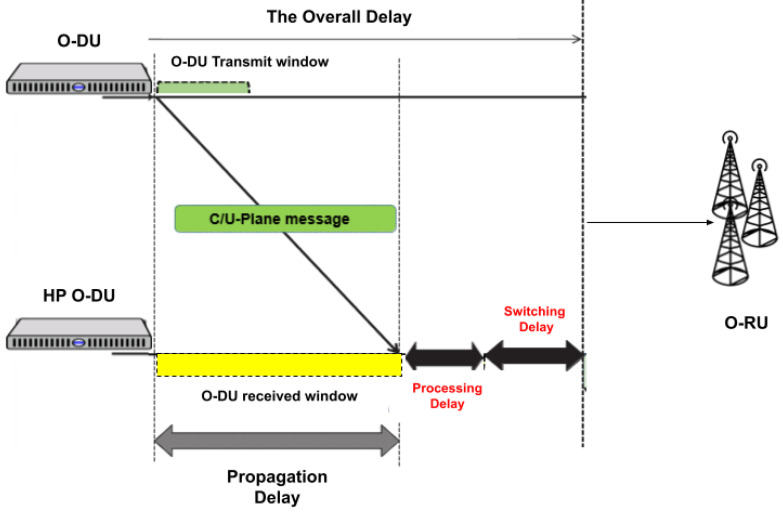
The overall delay in the network.

Since, in the O-RAN architecture, intelligence is integrated at multiple layers of O-RAN, it is natural to split the intelligence into different layers to take advantage of higher processing power or to be able to apply real-time actions for delay-sensitive applications. Accordingly, each O-RAN component should abide by a specific delay bound based on the tolerable delay within its control loop. To this end, in this work, we deploy the DU selection agent at CU to access a higher perspective with respect to DUs, accessing higher processing power to optimize the more complex problem and its higher tolerable delay bound (10 ms to 1 s) which provides adequate time for processing the model. Additionally, the RL-based scheduler agents are located at DUs to schedule UEs close to real-time time intervals (less than 10 ms) [32]. The tolerable delay bound at each layer closely depends on the midhaul/fronthaul bandwidth and the range. In this work, this delay is assumed as the AI feedback’s round trip time (RTT), and as shown in Figure 2, it includes processing, propagation, and switching delay over the XHaul. As it is discussed in [33], the maximum tolerable delay for an XHaul with a 3 Gbps bandwidth and 400 km range is at most 10 ms; therefore, in the system model, we considered the propagation delay in this range (2 ms to 5 ms) and left some room for switching delay to make it close to the real-life condition. It should be noted that the main focus of this work is on propagation delay, and we assumed the switching delay and processing delay constant by considering the constant bitrate for all DUs and defining the processing power of $DU^{HP}$ proportional to $DU^{LP}$ (the processing power of $DU^{HP}$ is considered two to four times higher than $DU^{LP}$ which makes it capable of processing up to four DUs’ load at the same time).

### 4.2. Soft Actor–Critic Energy Aware Dynamic DU Selection/NF Placement Scheme (SA2C-EADDUS)

In this work, a soft actor–critic approach is employed since it provides hierarchical control during the learning procedure. In actor–critic models, to reduce the gradient, the corresponding value function needs to be learned to update and assist the system policy. In the proposed scheme, based on the amount of energy consumed at each DU, the DU’s processing power, priority of traffic, and its delay tolerance level, the DU for executing the RL-based scheduling agent will be selected. This selection is also performed by an A2C agent. To this end, a neural network (NN) with three layers of neurons is employed. Furthermore, every TTI neurons’ weights are updated by interactions occurring between the actor and the critic. In the exploitation stage, the NN works as a non-linear function, and it will be tuned by updating the weights. During each state *s*, the main goal of SA2C agents is to maximize their received reward *r*, by applying more accurate actions, *a*, which can be obtained by action–value (Q(s,a)) and state–value (V(s)) functions. The action–value function is used to estimate the effect of applying actions during states. Consequently, estimation of the outcome is obtained by the state–value function. In this work, for improving the convergence and increasing the stability of the model (by reducing the overestimation bias), the soft actor–critic model (SA2C) is used. The overall architecture of SA2C is depicted in Figure 1. In the SA2C model, instead of evaluating action or state value functions, we just need to estimate V(s). Moreover, the error parameter in SA2C is a metric for examining the effect of performed action by considering the expected value V(s), which can be demonstrated by $A\left(s_{t},a_{t}\right)=Q\left(s_{t},a_{t}\right)-V\left(s_{t}\right)$. In addition, the SA2C model is a synchronous model (unlike asynchronous actor–critic models) which makes it more consistent and suitable for disaggregated implementations. In the proposed scheme, each of the actors and critics contain independent neural networks which are demonstrated as follows:

- The critic’s neural network is used to estimate the corresponding value function for aligning the actor’s actions. In the proposed scheme we used two critics to minimize the overestimation bias.
- The actor’s neural network is used to estimate proper action (choosing the best DU for executing NF) during each time interval.

The states of the proposed DU selection scheme are extracted from the environment through a tuple, $S_{t}=\left\{DU_{type},QCI,CQI,HOL_{delay}\right\}$, by which DUs’ location and their amount of available processing power can be identified by the DU’s type, traffic type and its priorities with a QoS class identifier (QCI) channel quality indicator (CQI) for examining signal strength, and the amount of time packets stay in the scheduler queue or head-of-the-line delay ($HOL_{delay}$), respectively. Meanwhile there are two independent actions which are generated by agents during each time interval. The action space of the A2C-based scheduler is the location of the assigned resource block in the RB map. Additionally, the action space of the DU selection agent is the DU type which should be capable to handle NFs (in our case, we only consider the placement of the A2C-based scheduler[] however, our scheme can be extended to other NFs in the 5G stack [26,28]). The DU selection agent needs to collaborate with the A2C-based RB allocation agent in a way that the overall energy consumption in the network is reduced, while the expected QoS metrics (delay and packet delivery ratio) of the A2C-based RB allocation agent can be met.

We consider two separate reward functions for our agents since their objectives and their action spaces are different. In this work, the reward function for the A2C-based scheduler is defined as follows as in [28]:

(14) $$Reward_{scheduler}=\beta R_{1}+\gamma R_{2}+\Phi R_{3},$$

(15) $$\;R_{1}=max(sgn(cqi_{k}-\frac{\sum_{i=0}^{I}cqi_{j}}{K}),0)$$

(16) $$\;R_{3}=sinc\left(\pi \lfloor \frac{Packet_{Delay}}{Traffic_{Budget}}\rfloor \right)$$

Here, $UE_{i}$ feedback is considered as $cqi_{i}$, $R_{2}$ is an extra reward of 1 which is given for URLLC traffic to prioritize, the traffic delay budget and packet HOL delay are presented as $Traffic_{Budget}$ and $Packet_{Delay}$, respectively. β, γ, and Φ are weighting/scaling factors. The scheduling agent consists of an actor and a critic. The actor is located at the BSs and is responsible for allocating resource blocks to UEs, and the critic is integrated into the DUs to inspect actors and improve their decisions. In this model, the reward function is defined based on the CQI feedback, the amount of time the packet stays in the scheduling queue, and the UEs’ satisfaction. To this end, the reward function is defined in a way that actors can receive the reward when the received SINR becomes higher (higher CQI), scheduling delay is reduced, and UEs’ satisfaction ratio is increased (mean delay should be less than the delay budget). Therefore, when the RB map filled by an actor causes an inter-cell interference, the received SINR will be reduced, and the mean delay will be increased, leading to the reduction of the reward and punishment for the agent, which can improve the learning process in agents.

We define the reward for the DU selection agent as follows:

(17) $$\;Reward_{DUselector}=\tau \left({R_{1}}^{\prime }+{R_{2}}^{\prime }\right)-\omega E^{TOT},$$

(18) $$\;{R_{1}}^{\prime }=sinc\left(\pi \left\lfloor \frac{\delta_{\langle i,t\rangle }^{S}+\delta_{\langle i,t\rangle }^{P}}{\Delta_{\langle i,t\rangle k}}\right\rfloor \right)$$

(19) $${R_{2}}^{\prime }=\frac{n_{URLLC}}{n_{tot}}sinc\left(\pi \left\lfloor \frac{\alpha \times (\delta_{\langle i,t\rangle }^{S}+\delta_{\langle i,t\rangle }^{P})}{\Delta_{\langle i,t\rangle k}})\right\rfloor \right)$$

Here, $n_{URLLC}$ shows the number of UEs which generate URLLC traffic. This can be obtained through the QCI value which is assigned by the EPS bearer, and it shows packet priorities, types and their delay budget [34]. In this reward function, sinc(π⌊⌋) output is a binary value which is used to produce discrete actions in each time interval (0 or 1). As it was explained previously, RUs select their local DUs to perform higher layer processing. Our system model includes one DU with higher processing power than the others. For energy efficiency, one would consider offloading NFs of local DUs to the high-processing power DU and switching the local DUs off. This may also allow expanding the inter-cell interference observation capability of agents. However, it is important to note that this approach would have an adverse impact on delay since local DUs can provide access with less propagation delay than the distant high-power DU site. This is in addition to scheduling delay. Moreover, $DU^{HP}$ has a limited processing capacity, and we cannot transfer all of DUs’ load to it. Therefore, in our reward function, we want to make sure during each time interval that we choose a proper DU (priority of its packet and propagation delay with respect to $DU^{HP}$) in a way that the overall delay which each packet experiences (scheduling delay ($\delta_{it}^{S}$) + propagation delay ($\delta_{it}^{P}$)) always remains below the predefined delay budget ($\Delta_{itk}$) (Equation (18)). Moreover, since URLLC UEs are delay sensitive, we need to make sure that the mean delay of URLLC traffic is kept as low as possible with respect to other UEs (Equation (19)). To this end, by increasing the α the overall delay ($\delta_{it}^{S}+\delta_{it}^{P}$) can be proportionally reduced with respect to the predefined delay budget. In addition, we want to ensure the total energy consumption ($E^{TOT}$) is reduced by a third term which is calculated by Equation (2). τ and ω are scalar weights which are defined based on the priority for enhancing the packet delivery ratio (${R_{1}}^{\prime }$), reducing the overall delay (${R_{2}}^{\prime }$), and reducing energy consumption in the network ($E^{TOT}$). As a final remark, in this algorithm, unlike our previous work [7], we improve our reward function by considering the propagation delay between O-RAN components, fixed energy consumption, and dynamic energy consumption with respect to DUs processing capacity.

## 5. Performance Evaluation

The SA2C-EADDUS scheme is implemented in ns3-gym, which is a framework where OpenAI Gym (a tool for using machine learning libraries) is integrated into ns3 [35,36]. The neural network of the proposed scheme is developed by Pytorch. In simulations, we assume the number of UEs is between 40 to 80, the UEs are randomly distributed, and they are associated to closets DU among 4 *DU^LP^*s. The URLLC UE ratio is 10%, and we employ numerology zero with 12 subcarriers, which has 14 symbols per subframe and 15 KHz subcarrier spacing. Furthermore, scheduling decisions are applied every TTI.

The SA2C-EADDUS performance is evaluated by considering different processing capacities of DUs and two different traffic types. We assume local DUs processing capacity cannot handle more than one RU’s functions. Additionally, we assume there is a DU with higher processing capacity ($DU^{HP}$) which is located far from the other RUs. $DU^{LP}$ can reduce its energy consumption by offloading its tasks to a DU with higher processing power. However, based on its location with respect to $DU^{HP}$, the propagation delay would vary.

We examine two processing capacity levels for $DU^{HP}$ to evaluate the effect of increasing the observation level of agents over network performance. We also consider two traffic types (live stream video as eMBB traffic and intelligent transport system (ITS) as URLLC traffic) with different delay budgets. It should be noted that when the overall delay of a packet (scheduling and propagation delay) is higher than the predefined delay budget, we assume the packet is dropped. In Table 1, QoS metrics of each traffic type is depicted.

We use the proposed MILP solution as a benchmark for SA2C-EADDUS. Furthermore, we compare SA2C-EADDUS with three baselines. The first baseline was proposed in our prior work [28]. The second baseline is based on a deep reinforcement learning (DRL) scheduler as in [26], and the last baseline is a heuristic method. We integrated these approaches to our energy-aware dynamic DU selection scheme to evaluate their performance with respect to the proposed scheme. In the following, we briefly present our baselines.

### 5.1. Delay and Priority Aware Actor–Critic RB Allocation Algorithm (A2C-RBA)

In our previous work [28], we implemented an actor–critic learning-based resource block scheduler where RBs are allocated to UEs by considering the delay budget and the priority of the user traffic. The following algorithm is developed for reconfigurable wireless networks where actions can be autonomously applied over the network. The A2C-RBA can adapt itself to the dynamicity of the environment to increase the utility of the available resources. However, the A2C-RBA algorithm is solely running at $DU^{LP}$, and it cannot take advantage of $DU^{HP}$.

### 5.2. Deep-Reinforcement Learning-Based Energy-Aware Dynamic DU Selection Scheme (DRL-EADDUS)

In [26], the main objective of DRL agents is allocating RBs in a way that the mean delay of packets be minimized. The authors in this work considered UEs with different packet request patterns, and they developed a deep-reinforcement learning algorithm to schedule RBs during each time interval. This work is integrated with the proposed framework to reduce the energy alongside the RB allocation in the network. We integrate this work into our framework to have a fair comparison with the proposed scheme.

### 5.3. Heuristic DU Selection Method (Heuristic-DUS)

In the heuristic method, decisions are made based on the propagation delay between local DUs and $DU^{HP}$ with respect to a predefined threshold value and type of packets. In this algorithm, traffic in local DUs will be transferred to $DU^{HP}$ when the propagation delay is below the predefined threshold and packets are not URLLC. Therefore, to reduce the packet drop ratio, if a packet’s scheduling delay is higher than the threshold or URLLC traffic is scheduled for the next TTI, RBs will be assigned locally. Additionally, when the packets are scheduled at $DU^{HP}$, RBs will be assigned by considering other DUs RB maps to reduce the interference in the network. It should be noted that assigning a proper threshold value can be challenging due to the high dynamicity of the network parameters. The proposed heuristic algorithm is illustrated in Algorithm 1.


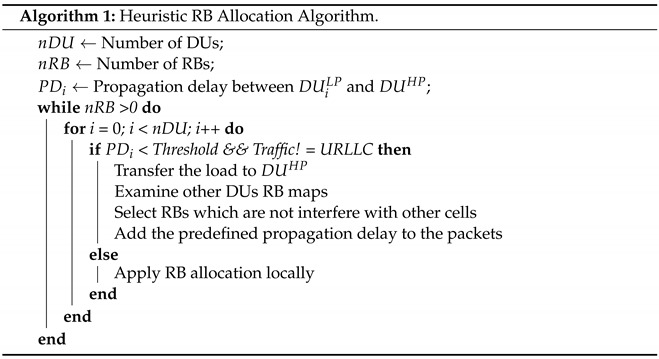


### 5.4. Simulation Results

In Table 2. the corresponding simulation and NNs parameters are illustrated in detail.

#### 5.4.1. Convergence

**Figure 3:**
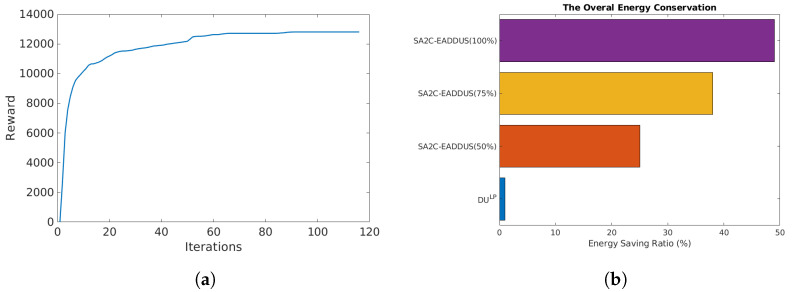
The convergence of the reward function and the overall energy conservation in the network when the SA2C-EADDUS with different processing capacity level is employed: (**a**) the convergence of the reward function; (**b**) impact of changing ξ in SA2C-EADDUS.

Before discussing the obtained results, in Figure 3a we present the convergence of the reward function for SA2C-EADDUS. In this figure, the number of UEs is equal to 70, of which 10% is assigned with URLLC traffic load. Additionally, we employed the epsilon-greedy policy, forcing actors to assign RBs with the highest weight or randomly choosing actions during the exploration phase. As it is shown, the algorithm will converge after almost 100 episodes where each episode contains 500 iterations.

#### 5.4.2. Energy Efficiency

As mentioned previously, in this work, the energy consumption is reduced by migrating a NF dynamically to a DU with a higher processing power by considering its available capacity, priority of packets, and propagation delay during each time interval. We assume that when a DU transfer its load to $DU^{HP}$, it can be deactivated, and its overall power consumption (dynamic + fix) will be zero during that time interval. We set the amount of fixed energy consumption at $DU^{LP}$ and $DU^{HP}$ as 5 KWh and 10 KWh, respectively [24]. We examine our model by considering three processing capacity levels for $DU^{HP}$. In the first one, we can transfer the processing load of all DUs ($DU^{LP}$) to $DU^{HP}$ (100%DRL-EADDUS), while in the second and third ones, $DU^{HP}$ has a limited processing power and the load of only 75% (75%-DRL-EADDUS) or 50% (50%-DRL-EADDUS) of $DU^{LP}$ can be transferred to $DU^{HP}$. As we can see in Figure 3b, based on the available processing power in $DU^{HP}$, by dynamically transferring local DUs’ load, the SA2C-EADDUS scheme can increase energy conservation up to 50%. Therefore, by dynamically transferring the load among DUs, we can reduce the energy consumption dramatically in the network.

As shown previously, the formulated problem is NP-hard. To this end, we first compare our approach with an optimization model in a smaller scale network, then in the following, we increase the size of the network and evaluate the proposed scheme in comparison to our baselines.

#### 5.4.3. Comparison with the MILP Solution

In this subsection, we compare the performance of our SA2C-EADDUS scheme with the MILP solution presented in Section 3.1. Due to the NP-hard property of our problem, we use only 12 RBs in this comparison. Therefore, we can obtain the optimum solutions with MILP solver in a reasonable run time.

**Figure 4:**
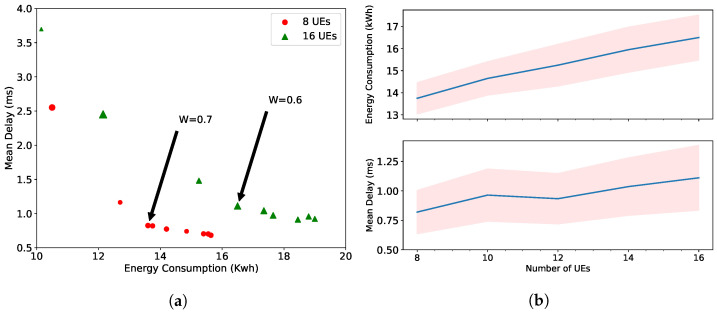
Energy consumption and mean delay performance with MILP solver: (**a**) the tradeoff between these two KPIs; (**b**) impact of increasing number of UEs.

Figure 4a shows the change of energy consumption and mean delay with the weighting factor of the energy consumption. As seen, W=0.7 and W=0.6 are the breaking points for 8 UEs and 16 UEs, respectively, in this multi-objective minimization problem. We observe that choosing a lower weight is crucial to prevent a drastically higher mean delay. In Figure 4b, we detail the impact of the increasing number of UEs when weight $W_{1}$ is set to =0.5. The mean delay remains in a reasonable range with the higher number of UEs, while the energy consumption increases due to the decentralization of the NFs. One of the main reasons for this decentralization comes from balancing the increase of scheduling delays due to increasing competition for RBs. Thus, NFs prefer to stay in local DUs to reduce the propagation delay, which also reduces the overall mean delay.

**Figure 5:**
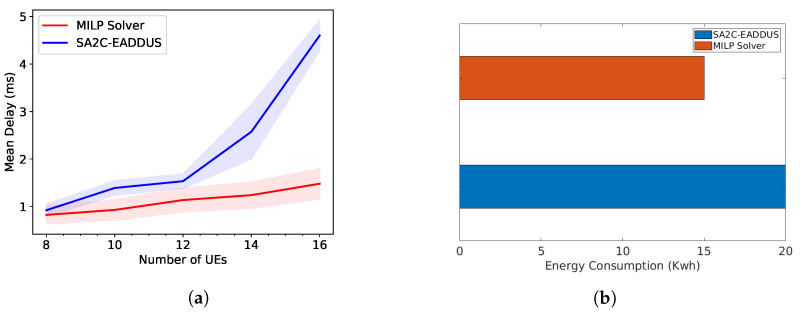
Lower bound comparisons of the proposed method SA2C-EADDUS: (**a**) mean delay comparison; (**b**) energy consumption comparison.

Figure 5a compares the mean delay results of the SA2C-EADDUS scheme and the MILP solution. The SA2C-EADDUS scheme provides a reasonable mean delay for the UEs lower than 14. However, due to the high contention, a higher number of UEs causes package drops, and then they cause the increase of mean delay. On the other hand, the MILP solver is not affected by this due to the ideal conditions and predefined given data. Figure 5b compares the energy consumption of two solutions.The SA2C-EADDUS scheme performs very close to the MILP solution.

#### 5.4.4. Delay

**Figure 6:**
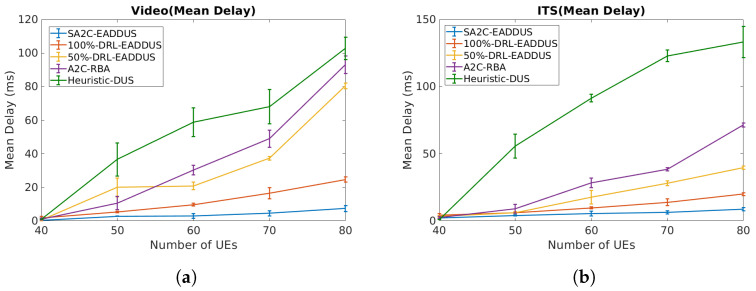
The overall mean delay by considering different processing capacity levels and algorithms: (**a**) the video packets’ mean delay; (**b**) the ITS packets’ mean delay.

Hereafter, we examine the performance of the SA2C-EADDUS scheme with respect to three baselines and use a larger network where the number of UEs are varied between 40 to 80. As shown in Table 1, we evaluated the proposed model by generating User Datagram Protocol (UDP) and Transmission Control Protocol (TCP) packets (video and ITS) with different delay budgets (150 ms and 30 ms, respectively) and QoS requirements. In Figure 6a,b we presented the mean delay of video packets and ITS packets independently to illustrate how the proposed model managed to keep the mean delay of each traffic type below its delay budget threshold. The proposed SA2C-EADDUS scheme is compared with an A2C-based scheduler (A2C-RBA) [28], a DRL-based scheduler with different processing capacity levels [26], and a heuristic scheme (Heuristic-DUS). Here, to examine the effect of the processing capacity of $DU^{HP}$ over the network performance, we assume that $DU^{HP}$ has two processing capacity levels. In the first one, we can transfer the processing load of all DUs ($DU^{LP}$) to $DU^{HP}$ (100%DRL-EADDUS), while in the second one, the load of only 50% of $DU^{LP}$ can be transferred to $DU^{HP}$ (50%-DRL-EADDUS). The energy consumption corresponding to the delay results in Figure 6 is depicted in Figure 3b. In Figure 3b, we presented the overall energy conversation when employing the SA2C-EADDUS with different capacity levels. The maximum energy will be consumed (20 KWh) when UEs are scheduled locally, and we can conserve energy consumption by up to 50% when the processing power increases.

As observed in Figure 6, the proposed algorithm reduces the mean delay in both ITS and video traffic with respect to baselines. Additionally, by increasing the processing power, the observation level of agents will be increased, and actions become more accurate; therefore, the 100%DRL-EADDUS agent performs better by reducing the mean delay in the network in comparison with the 50%-DRL-EADDUS agent. Finally, the SA2C-EADDUS can reduce the mean delay dramatically with respect to the A2C-RBA and the Heuristic-DUS algorithm, since the SA2C-EADDUS can allocate RBs in a way that the inter-cell interference in the network is reduced. Additionally, the SA2C-EADDUS approach performs better with respect to the DRL-EADDUS algorithm because, unlike DRL-EADDUS, the RB allocation agent in the SA2C-EADDUS scheme, in addition to packet delay, also considers the mean CQI level of UEs and the priority of URLLC packets in its algorithm.

#### 5.4.5. Packet Delivery Ratio

**Figure 7:**
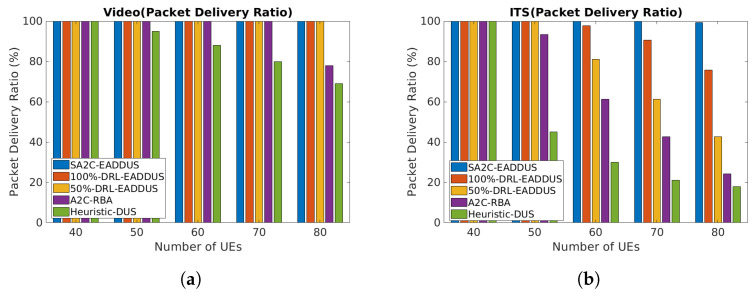
The overall packet delivery ratio by considering different processing capacity levels and algorithms: (**a**) the video packets’ delivery ratio; (**b**) the ITS packets’ delivery ratio.

As explained previously, the proposed scheme reduces the inter-cell interference in the network by expanding agents’ observation level and applying actions with respect to other RUs’ RB maps when the NFs are transferred to $DU^{HP}$. Therefore, as shown in Figure 7, the SA2C-EADDUS scheme can improve the packet delivery ratio significantly with respect to the A2C-RBA and Heuristic-DUS algorithms when the number of UEs are high. Similarly, by increasing the processing level of $DU^{HP}$, actions become more accurate, and the number of packets which are successfully delivered will increase.

## 6. Conclusions

As future mobile networks become more complex, the need for intelligence and participation of more players is emerging, eventually leading to the need for openness. As these goals are defining the initiatives such as O-RAN and several others, there is a dire need to explore intelligence capabilities. In this paper, we evaluated the significance of expanding the observation level in O-RAN architecture for NFs. To this end, we consider resource allocation function as an example NF and propose a two nested A2C-based algorithms, which contain two A2C techniques that are working together. The first layer dynamically transfers NFs to a DU with higher processing power to hit the balance between saving energy and improving actions’ accuracy. Meanwhile, the second layer contains an A2C-based scheduler algorithm, which allocates RB by considering user traffic type and their delay budget. The simulation results show that the proposed scheme can significantly increase energy efficiency and reduce the overall mean delay and packet drop ratio with respect to the case where the NF is solely executed at local DUs with limited processing power. In future works, we will employ the delay and energy consumption in the fronthaul links and the switching networks. 

## Figures and Tables

**Table 1:** Traffic properties [34].

QCI	Resource Type	Priority	Packet Delay Budget	Service Example
2	GBR	40	150 ms	Live stream Video
84	GBR	24	30 ms	ITS

**Table 2:** Simulation parameters.

Parameters	Value
Number of neurons	1024 × 512 layers (Actor + Critic)
DU selec./scheduler algorithms	SA2C-EADDUS, DRL-EADDUS, A2C-RBA
Number of BSs	4
Number of UEs	40, 60, 80
Maximum Traffic load per UE (Downlink)	512 kbps
Traffic types	Video, ITS
Propagation delay	2–5 ms
Reward’s weights	τ=0.5 and ω=0.5
Number of RBs	12, 100
Discount factor	0.9
Actor learning-rate	0.01
Critic learning-rate	0.05

## Notations

The following notations are used in this manuscript:

Sets	Explanation
r∈R	set of RBs in one time interval
t∈T	set of time intervals
i∈I	set of UEs
〈i,t〉∈〈I,T〉	tuple of demands
l∈L	set of $DU^{LP}$
k∈K	type of traffic
Variables	Explanation
$a_{\langle i,t\rangle rt^{\prime }}$	indicates $rt^{\prime }$ is assigned for 〈i,t〉
$b_{lt}$	indicates NFs of $DU^{LP}$ *l* migrates to $DU^{HP}$ in *t*
$y_{\langle i,t\rangle t^{\prime }}$	indicates any *r* in $t^{\prime }$ is assigned for 〈i,t〉
$u_{\langle i,t\rangle k}$	user traffic indicator
Given Data	Explanation
$D_{lt}^{P}$	propagation delay
$E_{l}^{F}$	fixed energy consumption at *l*
$E_{l}^{D}$	dynamic energy consumption coefficient at *l*
$E^{HP}$	total energy consumption at $DU^{HP}$
$S_{irt^{\prime }}$	max service rate
$U_{\langle i,t\rangle k}$	user traffic demand size
$\Delta_{\langle i,t\rangle k}$	delay budget
ξ	max. number of $DU^{LP}$ that can migrate their NFs
M(i)	user & $DU^{LP}$ map
TTI	length of transmission time interval
*W*	energy consumption weight in objective function
Ω	scaling factor between en. cons. and mean delay

## References

[1] Klinkowski M. (2020). Latency-Aware DU/CU Placement in Convergent Packet-Based 5G Fronthaul Transport Networks. Appl. Sci..

[2] Semov P., Koleva P., Tonchev K., Poulkov V., Cooklev T. (2020). Evolution of mobile networks and C-RAN on the road beyond 5G. Proceedings of the 2020 43rd International Conference on Telecommunications and Signal Processing (TSP).

[3] Dryjanski M., Kułacz Ł., Kliks A. (2021). Toward Modular and Flexible Open RAN Implementations in 6G Networks: Traffic Steering Use Case and O-RAN xApps. Sensors.

[4] Yi B., Wang X., Li K., Huang M. (2018). A comprehensive survey of network function virtualization. Comput. Netw..

[5] Gilson M., Mackenzie R., Sutton A., Huang J. (2018). NGMN Overview on 5G RAN Functional Decomposition.

[6] Pamuklu T., Ersoy C. (2021). GROVE: A Cost-Efficient Green Radio Over Ethernet Architecture for Next Generation Radio Access Networks. IEEE Trans. Green Commun. Netw..

[7] Mollahasani S., Erol-Kantarci M., Wilson R. Dynamic CU-DU Selection for Resource Allocation in O-RAN Using actor–critic Learning. Proceedings of the IEEE Global Communications Conference (GLOBECOM).

[8] Wu J., Zhang Y., Zukerman M., Yung E.K.N. (2015). Energy-efficient base-stations sleep-mode techniques in green cellular networks: A survey. IEEE Commun. Surv. Tutor..

[9] Oh E., Son K., Krishnamachari B. (2013). Dynamic base station switching-on/off strategies for green cellular networks. IEEE Trans. Wirel. Commun..

[10] Niu Z. (2011). TANGO: Traffic-aware network planning and green operation. IEEE Wirel. Commun..

[11] Mollahasani S., Onur E. (2019). Density-aware, energy-and spectrum-efficient small cell scheduling. IEEE Access.

[12] Qian M., Hardjawana W., Shi J., Vucetic B. (2015). Baseband processing units virtualization for cloud radio access networks. IEEE Wirel. Commun. Lett..

[13] Wang X., Thota S., Tornatore M., Chung H.S., Lee H.H., Park S., Mukherjee B. (2016). Energy-efficient virtual base station formation in optical-access-enabled cloud-RAN. IEEE J. Sel. Areas Commun..

[14] Sahu B.J., Dash S., Saxena N., Roy A. (2017). Energy-efficient BBU allocation for green C-RAN. IEEE Commun. Lett..

[15] Saxena N., Roy A., Kim H. (2016). Traffic-aware cloud RAN: A key for green 5G networks. IEEE J. Sel. Areas Commun..

[16] Malandrino F., Chiasserini C.F., Casetti C., Landi G., Capitani M. (2019). An Optimization-Enhanced MANO for Energy-Efficient 5G Networks. IEEE/ACM Trans. Netw..

[17] Larsen L.M., Checko A., Christiansen H.L. (2018). A survey of the functional splits proposed for 5G mobile crosshaul networks. IEEE Commun. Surv. Tutor..

[18] Shehata M., Elbanna A., Musumeci F., Tornatore M. (2018). Multiplexing gain and processing savings of 5G radio-access-network functional splits. IEEE Trans. Green Commun. Netw..

[19] Alabbasi A., Wang X., Cavdar C. (2018). Optimal processing allocation to minimize energy and bandwidth consumption in hybrid CRAN. IEEE Trans. Green Commun. Netw..

[20] Akoush S., Sohan R., Rice A., Moore A.W., Hopper A. (2010). Predicting the performance of virtual machine migration. Proceedings of the 2010 IEEE International Symposium on Modeling, Analysis and Simulation of Computer and Telecommunication Systems.

[21] Zhan Z.H., Liu X.F., Gong Y.J., Zhang J., Chung H.S.H., Li Y. (2015). Cloud computing resource scheduling and a survey of its evolutionary approaches. ACM Comput. Surv. (CSUR).

[22] Elsayed M., Erol-Kantarci M. (2019). AI-enabled future wireless networks: Challenges, opportunities, and open issues. IEEE Veh. Technol. Mag..

[23] Şahin T., Khalili R., Boban M., Wolisz A. (2018). Reinforcement learning scheduler for vehicle-to-vehicle communications outside coverage. Proceedings of the 2018 IEEE Vehicular Networking Conference (VNC).

[24] Pamuklu T., Erol-Kantarci M., Ersoy C. Reinforcement Learning Based Dynamic Function Splitting in Disaggregated Green Open RANs. Proceedings of the IEEE International Conference on Communications.

[25] Elsayed M., Erol-Kantarci M., Yanikomeroglu H. (2020). Transfer Reinforcement Learning for 5G-NR mm-Wave Networks. IEEE Trans. Wirel. Commun..

[26] Zhang T., Shen S., Mao S., Chang G.K. Delay-aware Cellular Traffic Scheduling with Deep Reinforcement Learning. Proceedings of the GLOBECOM 2020—2020 IEEE Global Communications Conference.

[27] Chen G., Zhang X., Shen F., Zeng Q. (2022). Two Tier Slicing Resource Allocation Algorithm Based on Deep Reinforcement Learning and Joint Bidding in Wireless Access Networks. Sensors.

[28] Mollahasani S., Erol-Kantarci M., Hirab M., Dehghan H., Wilson R. (2021). actor–critic Learning Based QoS-Aware Scheduler for Reconfigurable Wireless Networks. IEEE Trans. Netw. Sci. Eng..

[29] Pamuklu T., Mollahasani S., Erol-Kantarci M. Energy-Efficient and Delay-Guaranteed Joint Resource Allocation and DU Selection in O-RAN. Proceedings of the 5G World Forum (5GWF).

[30] O-RAN Alliance (2021). O-RAN-WG1-O-RAN Architecture Description—v04.00.00.

[31] Yu Y.J., Pang A.C., Hsiu P.C., Fang Y. (2013). Energy-efficient downlink resource allocation for mobile devices in wireless systems. Proceedings of the 2013 IEEE Global Communications Conference (GLOBECOM).

[32] Bonati L., D’Oro S., Polese M., Basagni S., Melodia T. (2021). Intelligence and Learning in O-RAN for Data-Driven NextG Cellular Networks. IEEE Commun. Mag..

[33] ITU (2018). ITU-T Recommendation G Suppl. 66. 5G Wireless Fronthaul Requirements in a Passive Optical Network Context.

[34] 3GPP (2020). Table 6.1.7-A: Standardized QCI Characteristics from 3GPP TS 23.203 V16.1.0.

[35] Gawłowicz P., Zubow A. NS-3 meets openai gym: The playground for machine learning in networking research. Proceedings of the 22nd International ACM Conference on Modeling, Analysis and Simulation of Wireless and Mobile Systems.

[36] Brockman G., Cheung V., Pettersson L., Schneider J., Schulman J., Tang J., Zaremba W. (2016). Openai gym. arXiv.

